# The ability of FGF21 to ameliorate signs of non-alcoholic fatty liver disease in mice depends on sex and diet

**DOI:** 10.18699/vjgb-26-88

**Published:** 2026-10

**Authors:** N.Yu. Balybina, E.N. Makarova, T.V. Jakovleva, A.Yu. Kazantseva, N.M. Bazhan

**Affiliations:** Institute of Cytology and Genetics of the Siberian Branch of the Russian Academy of Sciences, Novosibirsk, Russia; Institute of Cytology and Genetics of the Siberian Branch of the Russian Academy of Sciences, Novosibirsk, Russia; Institute of Cytology and Genetics of the Siberian Branch of the Russian Academy of Sciences, Novosibirsk, Russia; Institute of Cytology and Genetics of the Siberian Branch of the Russian Academy of Sciences, Novosibirsk, Russia; Institute of Cytology and Genetics of the Siberian Branch of the Russian Academy of Sciences, Novosibirsk, Russia

**Keywords:** non-alcoholic fatty liver disease (NAFLD), steatosis, FGF21, diet, obesity, sex differences, liver, неалкогольная жировая болезнь печени (НАЖБП), стеатоз, FGF21, диета, ожирение, половые различия, печень

## Abstract

Consumption of high-calorie food leads to the development of obesity and non-alcoholic fatty liver disease (NAFLD). The pathogenesis of NAFLD depends on the composition of the diet. Administration of fibroblast growth factor 21 (FGF21) to obese mice improves metabolism and liver condition; however, the dependence of this effect on sex and diet composition has not been studied. Objective: To investigate the influence of sex on liver condition in mice with diet-induced obesity caused by different diets and on the ability of FGF21 to beneficially affect liver function. Obesity was induced in male and female C57BL/6J mice using a high-fat diet (HFD) or a cafeteria diet (DCaf). After obesity development, mice were administered FGF21 (1 mg/kg for 7 days), and the morphofunctional state and gene expression in the liver were assessed. When maintained on an HFD, hepatic steatosis was less severe in females than in males. FGF21 did not affect the lipid droplet area in individuals of either sex but exerted a sex-dependent influence on gene expression: in females, it reduced the expression of glycolysis and lipogenesis genes, preventing lipid accumulation, while in males, it reduced the expression of inflammation, apoptosis, and fibrosis genes, preventing the transition from steatosis to steatohepatitis. When maintained on a DCaf, pronounced steatosis developed in mice of both sexes. FGF21 reduced liver lipid content regardless of sex. However, only in females did FGF21 reduce the expression of the Cidec gene involved in lipid droplet formation and increase the number of apoptotic bodies. In males, the effect of FGF21 on lipid accumulation in the liver appeared to be mediated by its action on other tissues. Thus, in obese mice, sex differences in the severity of hepatic steatosis depended on the diet type and were observed only against the background of HFD. Administration of FGF21 led to a reduction in the total area of lipid droplets in the liver in mice of both sexes only against the background of pronounced steatosis induced by the DCaf. The influence of FGF21 on gene expression in the liver was sex-dependent, both against the background of HFD and DCaf.

## Abstract (ru)

Потребление высококалорийной пищи вызывает развитие ожирения и неалкогольной жировой болезни печени (НАЖБП). Патогенез НАЖБП зависит от состава диеты. Введение фактора роста фибробластов 21 (FGF21) ожиревшим мышам улучшает метаболизм и состояние печени, однако зависимость этого эффекта от пола и состава диеты еще не изучена. Цель нашего исследования – изучить влияние пола на состояние печени у мышей с алиментарным ожирением, вызванным диетами разного состава, и на способность FGF21 благотворно влиять на метаболическую функцию печени. Ожирение индуцировали у самцов и самок мышей C57BL/6J высокожирной диетой (ВЖД) или диетой кафетерия (ДК). После развития ожирения мышам вводили FGF21 (1 мг/кг, 7 дней) и оценивали морфофункциональное состояние печени и экспрессию генов, участвующих в процессах метаболизма жиров и углеводов, воспаления, фиброза и апоптоза в ткани печени. При содержании на ВЖД стеатоз печени у самок был менее выражен, чем у самцов. FGF21 не влиял на площадь липидных капель у особей обоих полов, но оказывал зависящее от пола влияние на экспрессию генов: у самок снижал экспрессию генов гликолиза и липогенеза, препятствуя отложению липидов, а у самцов – генов воспаления, апоптоза и фиброза, предотвращая переход стеатоза в стеатогепатит (воспаление печени). При содержании на ДК у мышей обоих полов развивался выраженный стеатоз. FGF21 снижал содержание липидов в печени независимо от пола. Однако только у самок FGF21 снижал экспрессию гена Cidec, участвующего в образовании липидных капель, и повышал количество апоптозных телец. У самцов эффект FGF21 на накопление липидов в печени, по-видимому, опосредован действием на другие ткани: жировую, мышечную и т. д. Таким образом, у мышей с ожирением половые различия в степени выраженности стеатоза печени зависели от типа диеты и проявлялись только на фоне ВЖД. Введение FGF21 приводило к уменьшению общей площади липидных капель в печени у мышей обоих полов только на фоне выраженного стеатоза, вызванного содержанием на ДК. Влияние FGF21 на экспрессию генов в печени зависело от пола как на фоне высокожирной диеты, так и на фоне диеты кафетерия.

## Introduction

Obesity is a disease that leads to the development of a number of metabolic disorders, in particular non-alcoholic fatty liver disease (NAFLD). This disease is characterized by excessive lipid accumulation in the liver and has a progressive course ranging from hepatic steatosis (lipid deposition in hepatocytes) to non-alcoholic steatohepatitis (liver inflammation), fibrosis and cirrhosis (Friedman et al., 2018; Haas et al., 2019). The search for effective NAFLD treatment requires adequate laboratory models that reproduce the disorders occurring in humans.

One of the most common models of obesity and related diseases in mice is high-fat diet (HFD)-induced obesity (Speakman, 2019). On this type of diet, animals obtain 40 to 65 % of their calories from fat (Speakman, 2019). This diet promotes the development of pronounced obesity and associated disorders and is most often used in NAFLD research (Hu et al., 2018). However, in the human population, the main cause of obesity is more often the consumption of a combination of fats and carbohydrates. The corresponding model in laboratory animals is known as the “cafeteria diet” (DCaf) (Buyukdere et al., 2019). In this approach, animals are given a free choice between high-fat (lard, chips, sausages, etc.) and high‑carbohydrate food (cookies, biscuits, sweet drinks). It is assumed that the mechanisms of obesity development and associated disorders in animals are more accurately reproduced on a diet close to human diet pattern.

Sex influences the development of obesity. It has been shown that when mice are kept on an HFD, females usually have lower body and liver weight, as well as lower liver triglyceride content, than males (Senthil et al., 2014; Makarova et al., 2021b). The expression of the insulin receptor gene (InsR) and genes involved in lipid metabolism (Ppara, Pgc1a, Fgf21, Acca, Accb, Fasn) is higher in females compared to males (Makarova et al., 2021b). However, it is not known whether sex differences in the severity of NAFLD occur under these conditions. For the first time, the present work comprehensively investigates inflammation, fibrosis and apoptosis genes whose expression changes during the development of non-alcoholic fatty liver disease. When mice are kept on a DCaf, females show more pronounced signs of impaired liver morphology and function, including greater accumulation of lipid droplets and triglycerides, and higher expression levels of pro‑inflammatory factor genes than males (Gasparin et al., 2018). Based on this, therapeutic approaches to NAFLD treatment may depend on sex and diet type

One of the promising candidates for NAFLD treatment is fibroblast growth factor 21 (FGF21), a member of the fibroblast growth factor family with a wide range of metabolic functions (Li H. et al., 2013). Administration of exogenous FGF21 alleviates manifestations of hepatic steatosis (Leow et al., 2023), reduces obesity, insulin resistance and blood free fatty acid levels in both rodents and primates (Fisher, Maratos-Flier, 2016; Makarova et al., 2021b; Bazhan et al., 2023). These findings have stimulated the study of FGF21 as a potential therapy for NAFLD and other metabolic disorders.

It has been shown that in mice, the expression of endogenous FGF21 and the effects of recombinant FGF21 on carbohydrate and lipid metabolism depend on the sex of the animal and the etiology of obesity (Makarova et al., 2020, 2021a; Bazhan et al., 2023). Against the background of HFD, FGF21 improves general metabolic parameters in both sexes (Makarova et al., 2021b) and reduces NAFLD signs in males (Keinicke et al., 2020). However, the effect of FGF21 on NAFLD signs in females on HFD has not been studied. When animals are kept on a DCaf, FGF21 reduces blood fatty acid and leptin levels, improves glucose tolerance and increases the expression of genes involved in the regulation of lipid metabolism in the liver only in male mice (Bazhan et al., 2023). It can be assumed that on a DCaf, there is sexual dimorphism in the effects of FGF21 on NAFLD signs. This issue has not been investigated. The aim of this study is to investigate the effect of sex on liver condition in mice with diet-induced obesity caused by diets of different composition, and on the ability of FGF21 to beneficially influence liver metabolic function.

## Research methods

Animals. This work is a continuation of experiments assessing the effect of exogenous FGF21 on food choice and metabolic parameters in mice of different sexes with obesity induced by diets of different compositions. The experimental design and diet composition are described in detail in previously published studies (Makarova et al., 2021b; Bazhan et al., 2023). Two experiments were conducted using male and female C57BL/6J mice from the vivarium of the Institute of Cytology and Genetics SB RAS, Novosibirsk.

The experiment on the effect of FGF21 administration on metabolic parameters in mice with obesity induced by a high‑fat diet was performed in the SPF vivarium of the Institute of Cytology and Genetics SB RAS. To induce obesity, 12‑week‑old mice (17 males and 20 females) were given, in addition to standard chow (Ssniff Spezialdiäten GmbH, protein 19 %, fat 3.3 %), high‑fat diet pellets in which 60 % of calories come from fat (D12492, Ssniff Spezialdiäten GmbH, protein 24.4 %, fat 34.6 %). Thereafter, animals were kept on a mixture of these diets, with mice obtaining 80–90 % of their energy from the HFD (Makarova et al., 2021b). In this experiment, females gained weight less intensively than males. FGF21 administration was started when male body weight reached 44–46 g and female body weight exceeded 30 g. The age of mice at the start of FGF21 administration ranged from 28 to 38 weeks.

The experiment on the effect of FGF21 administration on metabolic parameters in mice with obesity induced by a cafeteria diet was performed in a conventional vivarium of the Institute of Cytology and Genetics SB RAS (Bazhan et al., 2023). To induce obesity, 10‑week‑old mice were given, in addition to standard chow (Assortiment Agro, Turakovo, Moscow Region, Russia), lard and sweet butter cookies. Previously, we showed that on such a diet, weight gain rates do not differ between females and males (Bazhan et al., 2023). FGF21 administration was started at the age of 28 weeks, when the average body weight of male mice was 42.13 g (range 36.1–47.5 g) and that of females was 41.6 g (range 37.3–44.2 g).

In each experiment, animals were divided into four groups: males receiving vehicle (phosphate‑buffered saline, PBS), males receiving FGF21, females receiving PBS, and females receiving FGF21. Each group contained 5–7 individuals.

For seven days, mice were subcutaneously injected with recombinant mouse FGF21 protein at a dose of 1 mg/kg body weight or an equivalent volume of PBS. The dose was selected based on the results of J. Xu et al., who administered FGF21 at doses of 0.1, 1, and 10 mg/kg and showed that the pharmacological effect of FGF21 begins to manifest at a dose of 1 mg/ kg body weight (Xu et al., 2009). At the end of the experiment, one day after the last injection, animals were decapitated. The liver was excised and weighed. Relative liver weight was calculated relative to lean body mass (body weight minus fat mass). For subsequent gene expression analysis, liver samples were frozen in liquid nitrogen. Liver samples for histological analysis were placed in 10 % formalin solution.

Production and purification of mouse recombinant FGF21 protein. Production and purification of mouse recombinant FGF21 were carried out according to a previously published protocol (Makarova et al., 2021b).

Determination of triglyceride levels in the liver. To determine triglyceride levels, liver samples were placed in PBS at a ratio of 8 mL PBS per 1 mg of sample. The sample was then homogenized and centrifuged at low speed (~50g). The supernatant was collected and centrifuged again at the same speed. After the second centrifugation, the supernatant was collected for triglyceride content determination. Triglyceride concentration was measured using an enzymatic colorimetric method with the “Triglycerides‑Novo liquid form” kit from Vector‑Best (cat. No. В‑8323).

Histological analysis of liver structure. To assess the degree of steatosis (lipid accumulation in the liver, as well as macrophage and apoptotic body content), a generally accepted diagnostic approach was used (Yanko et al., 2021; Leow et al., 2023). Liver sections were stained with hematoxylin and eosin according to the standard protocol (Makarova et al., 2021a). F.R.S. Gasparin et al. showed that the non‑stained round inclusions detected by hematoxylin‑eosin staining are lipid inclusions and that their number correlates with the amount of lipids determined by Sudan III staining (Gasparin et al., 2018).

For histological analysis of liver structure, a Zeiss light microscope (Axioskop 2 plus, Carl Zeiss Microscopy GmbH, Jena, Germany) with a built‑in camera was used. For each animal, 10 photographs of liver sections were taken at ×400 magnification. Statistical analysis was performed on averaged data for each animal (n = 5–7). The area of lipid droplets, the number of macrophages and the number of apoptotic bodies were quantified using ImageJ software (National Institutes of Health, Bethesda, MD, USA). Lipid droplets of different sizes were distinguished. Large lipid droplets were those that displaced the nucleus to the cell periphery; medium‑sized droplets were closely apposed lipid inclusions that did not displace the nucleus; and small lipid droplets were those located at a distance from each other. The total area occupied by lipids (sum of areas of lipid droplets of different sizes) and the area occupied by lipid droplets of different sizes relative to the total area of the photograph were calculated (Makarova et al., 2021a).

Assessment of mRNA levels of genes. The mRNA levels of genes involved in lipid and carbohydrate metabolism, inflammation, fibrosis and apoptosis were studied by relative quantitation using reverse transcription and real‑time PCR (relative quantitation real‑time PCR). RNA was isolated from tissue samples using ExtractRNA reagent for total RNA isolation (Eurogen, Moscow, Russia) according to the manufacturer’s instructions. Reverse transcription was performed using MMLV reverse transcriptase (Eurogen, Moscow, Russia) and oligo‑dT primer according to the manufacturer’s protocol. PCR was performed according to the instructions using a ready‑made reaction mixture qPCRmix‑HS LowROX (Eurogen, Moscow, Russia) and reagents from Applied Biosystems: TaqMan Gene Expression Assay for the mouse genes Beta‑actin, Actb, Mm00607939_s1C. The expression of genes involved in various processes and systems of the liver was examined: glycolysis (Glucokinase, Gck, Mm00439129_m1; Pyruvate kinase liver and red blood cell, Pklr, Mm00443090_m1); gluconeogenesis (Glucose‑6‑phosphatase, G6p, Mm00839363_m1; Phosphoenolpyruvate carboxykinase 1, Pck1, Mm01247058_ m1); FGF21 system (Fibroblast growth factor 21, Fgf21, Mm00840165_g1; Klotho beta, Klb, Mm00473122_m); lipid oxidation (Peroxisome proliferator activated receptor alpha, Ppara, Mm0040939_m1; Carnitine palmitoyltransferase 1a, Cpt1a, Mm01231183_m1; Peroxisome proliferative activated receptor, gamma, coactivator 1 alpha, Ppargc1a, Mm01208835_m1); lipid synthesis (Fatty acid synthase, Fasn, Mm00662319_m1; Acetyl‑coenzyme A carboxylase alpha, Acca, Mm01304285_m1; Acetyl‑coenzyme A carboxylase beta, Accb, Mm01204683_m1; Cell death‑inducing DFFA‑like effector c, Cidec, Mm00617672_m1); inflammation and fibrosis (Tissue inhibitor of metalloproteinase 1, Timp1, Mm01341361_m1; Transforming growth factor, beta 1, Tgfb1, Mm01178820_m1; CD68 antigen, Cd68, Mm03047343_m1; Tumor necrosis factor a, Tnfa, Mm00443258_m1; Interleukin 1 beta, Il1b, Mm00434228_m1; Nuclear factor, erythroid derived 2, like 2, Nfe2l2, Mm00477784_m1; Chemokine (C‑C motif) ligand 3, Ccl3, Mm00441259_g1); apoptosis (BCL2, apoptosis regulator, Bcl2, Hs04986394_s1; BCL2‑antagonist/ killer 1, Bak1, Mm00432045_m1); growth factor (Insulin‑like growth factor 1, Igf1, Mm00439560_m1). Beta‑actin was used as an endogenous control. PCR and fluorescence detection were performed on an Applied Biosystems VIIA 7 real‑time PCR system. Relative quantification was performed using the comparative threshold cycle (CT) method (2−ΔΔCt).

Statistical analysis. To analyze the effect of FGF21 on lipid accumulation in the liver, two‑way ANOVA with the factors “sex” and “experimental group” (EG) was used. For multiple comparisons, the post‑hoc Newman–Keuls test was used. Results were considered statistically significant at p < 0.05.

Due to heterogeneity of variances, gene expression data were analyzed using the non‑parametric Mann–Whitney U test (n = 5–7). Significance was set at p < 0.05. Gene expression data are presented as medians, means, maximum and minimum values, as well as the 25–75 % range of values for each parameter.

All analyses were performed using the STATISTICA 6 software package (StatSoft, TIBCO Software Inc., Palo Alto, CA, USA).

## Results

High‑fat diet experiment. Liver morphology. Figure 1a shows a typical histological picture of the liver in obesity: hepatocytes containing large, medium and small lipid droplets, as well as macrophages and apoptotic bodies.

In animals kept on a high‑fat diet, signs of steatosis in the liver of females were less pronounced than in males (Fig. 1b). Relative liver weight was lower in females than in males (effect of the factor “Sex”, F(1.20) = 7.52, p < 0.05) (Fig. 2a). The area of small lipid droplets and liver triglyceride content in PBS‑treated females and males did not differ, whereas the total lipid droplet area (effect of “Sex”, F(1.20) = 7.45, p <0.05) and the area of medium‑sized (F(1.20) = 12.39, p <0.01) and large (F(1.20) = 9.93, p <0.01) droplets were significantly lower in females than in males (Fig. 2b, c, e). However, the number of macrophages (effect of “Sex”, F(1.20) = 39.07, p < 0.001; Newman–Keuls post‑hoc test, p < 0.05) and the number of apoptotic bodies (effect of “Sex”, F(1.20) = 5.02, p < 0.05; Newman–Keuls test, p < 0.05) were higher in females than in males, indicating more pronounced inflammation in females compared to males (Fig. 2d, e).

**Fig. 1.**
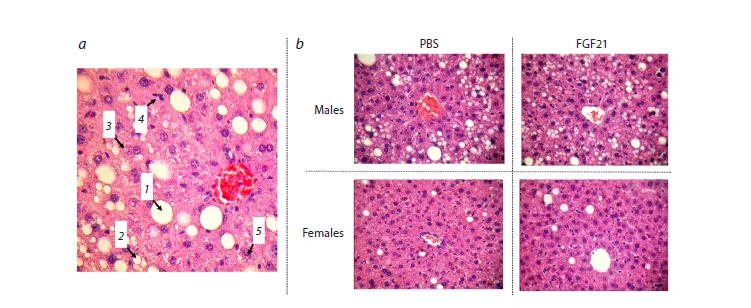
Representative images: a – lipid accumulation in the liver of male and female mice fed a high fat diet and receiving PBS or FGF21; b – general liver architecture in obese mice. Hematoxylin and eosin staining, ×400. 1 – large lipid droplets, 2 – medium lipid droplets, 3 – small lipid droplets, 4 – macrophages, 5 – apoptotic bodies.

**Fig. 2.**
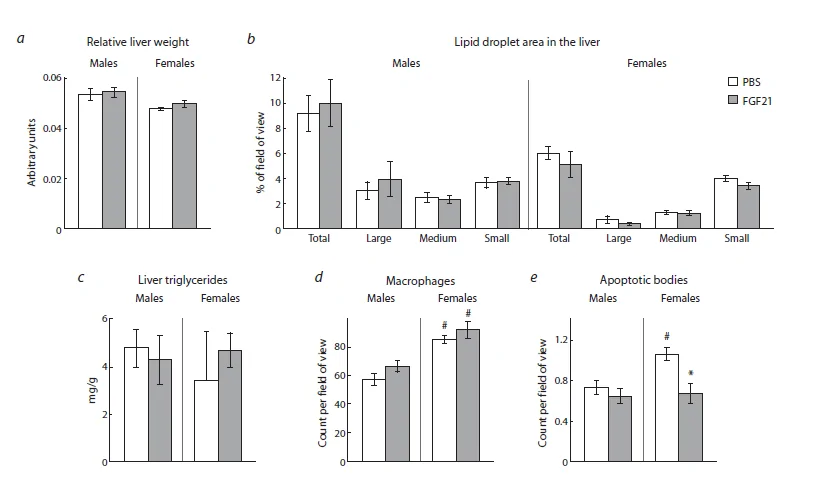
Relative liver weight (a), lipid droplet area (b), triglyceride content (c), macrophage count (d) and apoptotic body count (e) in the liver of male and female mice fed a high fat diet and receiving PBS or FGF21. # p < 0.05 vs. males, post-hoc Newman–Keuls test; * p <0.05 vs. PBS, post-hoc Newman–Keuls test.

FGF21 administration did not affect relative liver weight, triglyceride content, macrophage number or lipid droplet area in the liver, but reduced the number of apoptotic bodies (effect of the factor “EG”, F(1.20) = 8.67, p < 0.01; Newman–Keuls test, p < 0.05 for females) in the liver of both sexes (Fig. 2).

**Expression of genes involved in lipid and carbohydrate metabolism**

In HFD‑fed females treated with PBS, the expression levels of Fgf21 ( p <0.01, U = 0.00, n♀ = 5, N♂ = 7), Acca (p < 0.01, U = 0.00, n♀ = 5, N♂ = 7) and Ppara ( p < 0.05, U = 4.00, n♀ = 5, N♂ = 7) were higher than in males. FGF21 administration reduced the expression of Gck (p < 0.05, U = 3.00, n = 5 for both groups), Pklr ( p < 0.05, U = 2.00, n = 5 for both groups) and Fasn ( p <0.05, U = 2.00, n = 5 for both groups) in females and had no effect on the expression of the studied genes in males (Fig. 3).

**Fig. 3.**
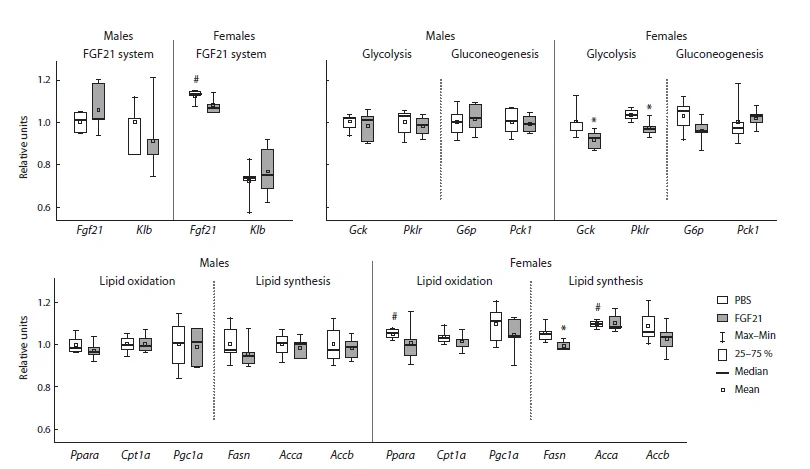
Relative expression levels of carbohydrate and lipid metabolism genes in the liver of male and female mice fed a high fat diet and receiving PBS or FGF21. # p < 0.05 vs. males, Mann–Whitney U test; * p <0.05 vs. PBS, Mann–Whitney U test.

**Expression of genes involved in inflammation, fibrosis and apoptosis**

In PBS‑treated animals, the expression levels of Tnfa ( p <0.05, U = 5.00, n♀ = 5, N♂ = 7) and Nfe2l2 ( p < 0.05, U = 2.00, n♀ = 5, N♂ = 7) were higher in females than in males. FGF21 administration reduced the expression of Tgfb1 ( p < 0.05, U = 6.50, n = 7 for both groups), Nfe2l2 ( p < 0.05, U = 9.00, n = 7 for both groups), Bak1 ( p <0.05, U = 2.00, n = 7 for both groups) and Igf1 ( p <0.05, U = 5.00, n = 7 for both groups) in males and had no effect on the expression of the studied genes in females (Fig. 4).

**Fig. 4.**
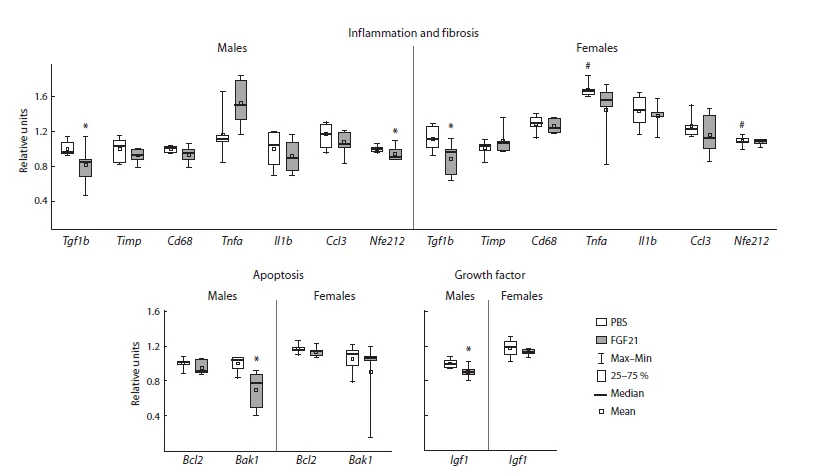
Relative expression levels of inflammation, fibrosis, apoptosis and insulin like growth factor genes in the liver of male and female mice fed a high fat diet and receiving PBS or FGF21. # p <0.05 vs. males, Mann–Whitney U test; * p <0.05 vs. PBS, Mann–Whitney U test.

Cafeteria diet experiment. Liver morphology. In animals kept on a cafeteria diet, control mice of both sexes showed high lipid content in the liver, with no sex differences in this parameter (Fig. 5 and 6b). Relative liver weight and liver triglyceride content did not differ between male and female mice (Fig. 6a, c). Females had an increased number of macrophages compared to males (effect of the factor “Sex”, F(1.14) = 4.56, p <0.05) (Fig. 6d). The number of apoptotic bodies in controls did not differ between females and males (Fig. 6e).

**Fig. 5.**
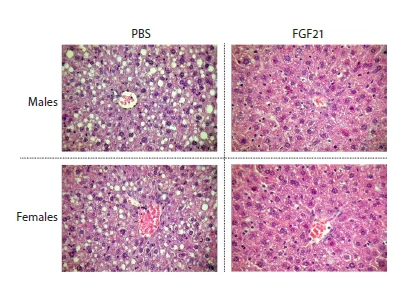
Representative images. Lipid accumulation in the liver of male and female mice fed a cafeteria diet and receiving PBS or FGF21. Hematoxylin and eosin staining, ×400.

**Fig. 6.**
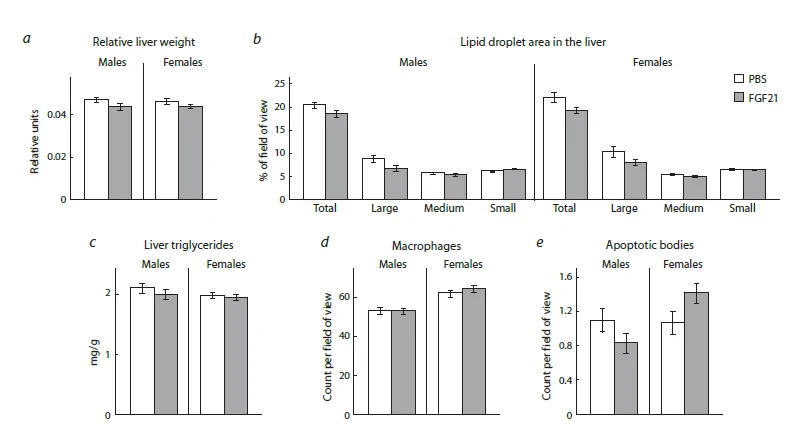
Relative liver weight (a), lipid droplet area (b), triglyceride content (c), macrophage count (d) and apoptotic body count (e) in the liver of male and female mice fed a cafeteria diet and receiving PBS or FGF21.

FGF21 administration reduced relative liver weight (effect of “EG”, F(1.14) = 4.94, p < 0.05) and total lipid droplet area in both males and females (effect of “EG”, F(1.14) = 4.68, p < 0.05) (Fig. 6a, b). FGF21 administration did not affect liver triglyceride content, macrophage number or apoptotic body number in either sex (Fig. 6c–e).

**Expression of genes involved in lipid and carbohydrate metabolism**

In obesity induced by the DCaf, only Cidec expression was lower in females ( p < 0.05, U = 3.00, n = 5 for both groups) than in males, and FGF21 reduced its expression only in females ( p <0.01, U = 0.00, n = 5 for both groups) (Fig. 7). No effect of sex or FGF21 was detected on the expression of other studied genes involved in the regulation of lipid and carbohydrate metabolism.

**Fig. 7.**
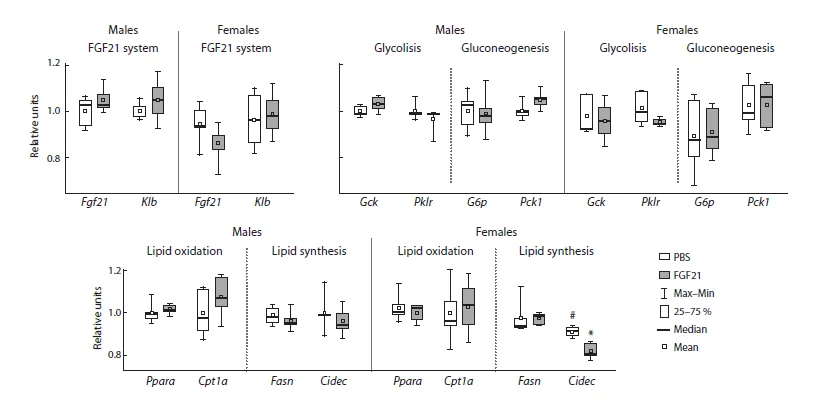
Relative expression levels of carbohydrate and lipid metabolism genes in the liver of male and female mice fed a cafeteria diet and receiving PBS or FGF21. # p < 0.05 vs. males, Mann–Whitney U test; * p < 0.05 vs. PBS, Mann–Whitney U test.

**Expression of genes involved in inflammation, fibrosis and apoptosis**

No sex differences were found in the expression levels of inflammation and fibrosis genes in the liver under DCaf‑induced obesity, nor any effect of FGF21 administration on the expression of these genes (Fig. 8).

**Fig. 8.**
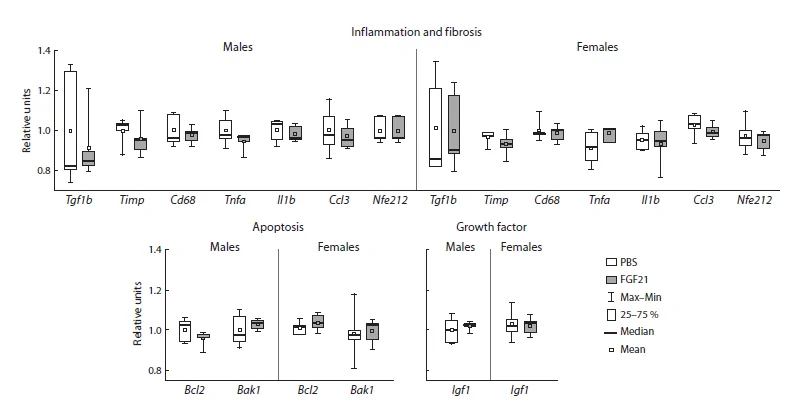
Relative expression levels of inflammation, fibrosis, apoptosis and insulin like growth factor genes in the liver of male and female mice fed a cafeteria diet and receiving PBS or FGF21.

## Discussion

In this study, we investigated sex‑specific features of steatosis development in diet‑induced obesity caused by diets of different composition, as well as the effect of FGF21 on the morphofunctional state of the liver and on the expression of genes involved in carbohydrate and lipid metabolism, inflammation, fibrosis and apoptosis in the liver of male and female mice under different types of obesity.

A high‑fat diet is widely used to study the mechanisms of obesity development and potential agents for correcting associated disorders (Li J. et al., 2020). It has previously been shown that HFD consumption promotes the development of obesity and non‑alcoholic fatty liver disease in male mice (Lau et al., 2017). In our study, we used a mixed diet: standard chow together with high‑fat pellets in which 60 % of calories come from fat. According to S. Hu et al., such nutrition in male mice causes more pronounced disturbances in metabolic parameters, faster obesity development, a significant decrease in insulin sensitivity and greater liver fat deposition compared to diets in which 10 and 45 % of calories come from fat (Hu et al., 2018).

HFD consumption revealed significant sex differences in liver histology. Relative liver weight and the area occupied by lipid droplets were significantly lower in females than in males, indicating less severe steatosis (Fig. 1 and 2). Previously, other authors (Hwang et al., 2010) and ourselves in this experiment (Makarova et al., 2021b) showed that on an HFD, female mice exhibit a lower degree of whole‑body obesity, manifested by lower body weight, lower fat mass and lower liver weight compared to males.

Decreased insulin sensitivity promotes lipid deposition in the liver and NAFLD development (Takaki et al., 2013). In the present experiment (Makarova et al., 2021b) and by other authors (Hwang et al., 2010), female mice were shown to be more insulin‑sensitive than males; therefore, the lower lipid accumulation in the liver of females may be a consequence of their higher insulin sensitivity.

The lower lipid accumulation in the liver of females on HFD may also be explained by higher lipid metabolism activity. The expression levels of genes encoding lipogenesis enzymes (Acca) and fatty acid oxidation enzymes (Ppara) were higher in females (Fig. 3). Females also had increased expression of the Fgf21 gene in the liver. FGF21 can trigger the expression of genes involved in free fatty acid oxidation, thereby reducing lipid deposition in the liver (Kleiner et al., 2012; Fisher, Maratos-Flier, 2016).

On HFD, females exhibited a higher expression level of the pro‑inflammatory factor gene Tnfa and a lower expression level of the Nfe2l2 gene, which encodes a transcription factor that protects cells from oxidative stress by activating genes involved in antioxidant and detoxification reactions (Fig. 4). It has been shown that under prolonged stress, its level may decrease, exacerbating signs of oxidative stress (Ye et al., 2023). These facts indicate that, despite lower fat deposition in the liver, signs of oxidative stress were more pronounced in females than in males.

We observed in HFD‑fed females a greater number of macrophages and apoptotic bodies in the liver parenchyma compared to males, both after PBS and FGF21 administration (Fig. 2). Similar changes were observed in genetic melanocortin obesity (Makarova et al., 2021a). Macrophages, in turn, express pro‑inflammatory cytokines such as Il1b, Tnfa and Il6, which promote the progression of inflammation and the development of steatohepatitis (Alkhouri et al., 2011).

Thus, we show for the first time that when consuming high‑fat food, females demonstrate lower accumulation of lipid droplets in the liver compared to males. However, they show higher expression of the Tnfa gene and greater numbers of macrophages and apoptotic bodies, which may indicate a higher degree of inflammation or may represent an adaptive response to high‑fat diet consumption in female mice. This question requires further investigation.

When FGF21 was administered to HFD‑fed mice, we observed suppression of glycolysis (Pklr, Gck) and lipogenesis (Fasn) gene expression only in females (Fig. 3), which may indicate the activation of adaptive mechanisms protecting the liver from excessive fat deposition. Despite this, no reduction in lipid droplet numbers was found in females. We attribute this to the fact that, according to the literature, the baseline lipid level in females was only slightly elevated compared to normal (Kim et al., 2013), which limited the scope for visible improvement.

In HFD‑fed males, which had more severe steatosis than females, FGF21 also did not affect liver lipid content (Fig. 2). The lack of effect may be explained by the fact that FGF21 suppressed Igf1 gene expression in the liver (Fig. 4) (Boparai et al., 2015). It is known that IGF1 prevents fat accumulation in the liver (Ma, Stanley, 2023). Possibly, the inhibitory effect of FGF21 on Igf1 expression, observed by us and other researchers, may counteract the direct lipid‑lowering action of IGF1.

On HFD, both sexes showed a decrease in the number of apoptotic bodies in the liver in response to FGF21 administration (Fig. 2), and males also showed decreased expression of the pro‑apoptotic gene Bak1 (Fig. 5). Since inflammation is often associated with apoptosis, these changes may indicate reduced liver inflammation (Chen et al., 2018). Moreover, in males, FGF21 reduced the expression of the pro‑fibrotic gene Tgfb1, which may prevent the development of fibrosis – a characteristic sign of NAFLD progression.

Thus, in obesity induced by a high‑fat diet, the effect of FGF21 on liver status depended on the degree of steatosis: when lipid content was low (as in females), the effect was aimed at preventing fat deposition by suppressing glycolysis and lipogenesis gene expression. When steatosis was pronounced (as in males in this study), the effects of FGF21 were directed towards reducing the expression of genes involved in the inflammatory process.

Previously, we and other authors have shown that when excess carbohydrates are added to the diet together with excess fat, females achieve a greater degree of obesity than males (Gasparin et al., 2018; Bazhan et al., 2020). However, in the present study we found that the area of lipid droplets in the liver did not differ between males and females (Fig. 6). The absence of differences in liver lipid accumulation despite greater obesity in females on the DCaf may be related to their relatively lower expression of the Cidec gene compared to males (Fig. 7). The product of this gene plays a key role in lipid droplet formation in the liver. It has been shown that animals with reduced Cidec expression exhibit a marked reduction in lipid stores in various tissues (Chen et al., 2018).

In obesity induced by the DCaf, females had more macrophages in the liver than males, which may indicate that their liver obesity is complicated by inflammation (Fig. 6). Previously, F.R.S. Gasparin et al. (2018) and A. Spruss et al. (2012) observed more pronounced signs of liver inflammation in females than in males. Thus, in animals kept on a cafeteria diet, no differences were found in the degree of hepatic steatosis, but females showed signs of greater liver inflammation than males.

Administration of FGF21 to DCaf‑fed mice led to a decrease in relative liver weight and hepatocyte lipid content in both sexes (Fig. 6). The mechanism of this effect differed: in females, FGF21 suppressed the expression of the Cidec gene in the liver, whose product plays a key role in the formation and fusion of lipid droplets (Fig. 7) (Chen et al., 2018). In DCaf‑fed males, FGF21 did not affect the expression of liver genes involved in the regulation of lipid metabolism. It can be assumed that in males, the FGF21‑induced reduction in lipid droplet area in the liver was related to its action on white adipose tissue, which is the main target organ for FGF21 (Makarova et al., 2021b). Previously, we showed that in male mice with DCaf‑induced obesity, FGF21 reduces blood free fatty acid levels (Bazhan et al., 2023). This may reduce fat uptake by the liver and its subsequent deposition as lipid droplets.

Thus, FGF21 had a similar effect on liver lipid content in male and female C57BL/6J mice on a cafeteria diet. However, the mechanisms underlying this effect differed: in females it likely acted through suppression of a lipogenesis gene in the liver, whereas in males it acted through extrahepatic mechanisms.

It is known that sex differences in the metabolic effects of FGF21 depend on the cause of obesity. Thus, on a high‑fat diet, FGF21 exerts unidirectional effects in males and females on body weight and fat mass, blood cholesterol, glucose and insulin levels, and on the expression of glycolysis and fatty acid synthesis genes in the liver (Makarova et al., 2021b). In animals kept on a cafeteria diet, the therapeutic effects of FGF21 administration are more pronounced in males – manifested as reduced plasma fatty acid and leptin levels, improved glucose tolerance, and increased expression of genes involved in insulin signaling and lipid metabolism in the liver (Bazhan et al., 2023).

In the present study, we found that the degree of hepatic steatosis depends on the type of diet offered to the animals. Sex differences in liver lipid accumulation were observed in the HFD model, which was characterized by early stages of hepatic steatosis in mice. On HFD, female mice showed a small amount of small lipid droplets with virtually no large or medium‑sized droplets; the total lipid droplet area in females was about 6 % of the field of view, corresponding to a slight increase above the normal level in mice (normal ~5 %) (Leow et al., 2023). In contrast, male mice had lipid droplets of all sizes in the liver, and the total area exceeded 9 % of the field of view, corresponding to the initial stages of the first (mild) stage of hepatic steatosis in mice (Leow et al., 2023) (Fig. 9).

**Fig. 9.**
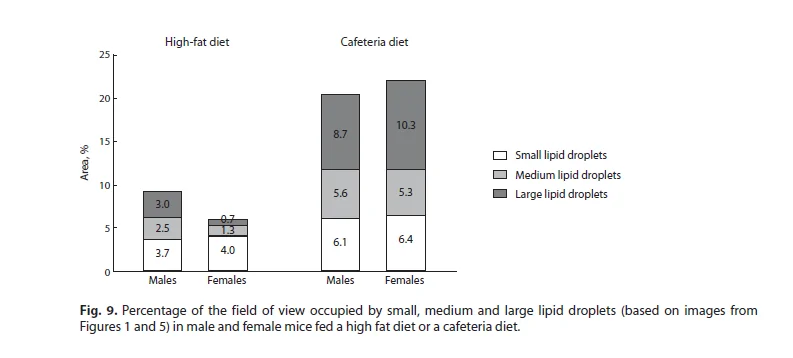
Percentage of the field of view occupied by small, medium and large lipid droplets (based on images from Figures 1 and 5) in male and female mice fed a high fat diet or a cafeteria diet.

DCaf consumption caused more pronounced accumulation of lipid droplets of various sizes in the liver of both sexes than HFD consumption, with no sex differences in the degree of steatosis. In both males and females, the total lipid droplet area exceeded 20 %, corresponding to a later stage of the first stage of hepatic steatosis (Leow et al., 2023) (Fig. 9).

The differences we observed in liver lipid accumulation between diet types may be due to several factors. The DCaf provides not only fats but also an excess of simple carbohydrates (sugars); consumption of simple carbohydrates activates specific de novo lipogenesis pathways, which can operate in parallel with dietary fat assimilation (Sanders, Griffin, 2016). This creates an additional burden on lipid metabolism. Moreover, an excess of carbohydrates in the diet promotes the development of insulin resistance, which in turn leads to fat accumulation in the liver (Takaki et al., 2013). In addition, the DCaf may cause more pronounced oxidative damage to liver cells due to the combination of different types of fats and excess sugars, disrupting lipid utilization processes and promoting steatosis development (Asrih, Jornayvaz, 2013).

The effect of FGF21 on the liver depended on the degree of steatosis. In female mice on HFD, where steatosis was not pronounced, the effect of FGF21 was to prevent lipid accumulation in the liver by reducing the expression of glycolysis and lipogenesis genes. In male mice on the same diet, but with early‑stage steatosis, the effect of FGF21 was to suppress the expression of inflammation and fibrogenesis genes. It is known that already at the early stages of NAFLD development, the expression of pro‑inflammatory cytokines increases, which contributes to disease aggravation (Asrih, Jornayvaz, 2013). Apparently, the main action of FGF21 in this case is aimed at preventing the development of non‑alcoholic steatohepatitis.

At the more advanced stage of hepatic steatosis observed in both male and female mice on DCaf, the effect of FGF21 was directed towards reducing the area of lipids in the liver. However, the mechanism of this effect differed between males and females: in females, a decrease in the expression of the Cidec gene, which plays a key role in lipid droplet formation and fusion (Chen et al., 2018), was observed, whereas in males no direct effect of FGF21 on the liver was detected.

## Conclusion

The present study revealed sex differences in the development of non‑alcoholic fatty liver disease and in the response to FGF21 therapy depending on the type of diet. On a high‑fat diet, females displayed less pronounced steatosis, which was associated with higher expression levels of lipid metabolism genes and higher basal Fgf21 expression. However, judging by the expression of inflammation genes and the number of macrophages and apoptotic bodies in the liver, steatosis in females was accompanied by an enhanced inflammatory response, increasing the risk of steatohepatitis. In contrast, on a cafeteria diet, steatosis was equally pronounced in both sexes, with more pronounced signs of inflammation in females compared to males.

The effects of FGF21 depended on sex and the stage of steatosis: at early stages of steatosis (high‑fat diet) in females, judging by Gck and Pklr expression, it suppressed glycolysis (both genes belong to glycolysis) and lipogenesis (Fasn), preventing lipid accumulation, whereas in males it reduced signs of inflammation and exerted an anti‑fibrotic action. At a more advanced stage of steatosis (cafeteria diet), FGF21 reduced liver lipid content in both male and female mice, but by different mechanisms – in females through suppression of Cidec gene expression, in males indirectly, apparently through action on adipose tissue. Thus, the therapeutic strategy for using FGF21 in non‑alcoholic fatty liver disease requires consideration of the patient’s sex, the stage of steatosis and the etiology of obesity.

Ethics approval. The study was conducted according to the guidelines of the Declaration of Helsinki and approved by the Independent Ethics Committee of the Institute of Cytology and Genetics, Siberian Division, Russian Academy of Sciences (Protocol No. 47 dated February 23, 2019, for HFD (high-fat diet), and dated November 8, 2021, for DCaf (cafeteria diet).

## Conflict of interest

The authors declare no conflict of interest.
